# Moyamoya syndrome and neurofibromatosis type 1

**DOI:** 10.1186/1824-7288-40-59

**Published:** 2014-06-21

**Authors:** Euthymia Vargiami, Evdoxia Sapountzi, Dimitris Samakovitis, Spyros Batzios, Maria Kyriazi, Athanasia Anastasiou, Dimitrios I Zafeiriou

**Affiliations:** 1 1st Department of Pediatrics, Aristotle University of Thessaloniki, Egnatia St. 106, Thessaloniki 54622, Greece; 2 Department of Radiology, “Hippokratio” General Hospital, Thessaloniki, Greece

**Keywords:** Moyamoya syndrome, Neurofibromatosis type 1 (NF1), Vasculopathy, Infarct, Central nervous system, Childhood

## Abstract

Neurofibromatosis type 1 (NF1) is the most prevalent autosomal dominant genetic disorder among humans. NF1 vasculopathy is a significant but underrecognized complication of the disease, affecting both arterial and venous blood vessels of all sizes. Moyamoya syndrome is a cerebral vasculopathy that is only rarely observed in association with NF1, particularly in the pediatric age range. Herein, we report of a 5-year-old female with NF1 and moyamoya syndrome and we briefly review the existing literature.

## Introduction

Neurofibromatosis type 1 (NF1) or von Recklinghausen is the most common neurocutaneous contidion with an autosomal dominant pattern of inheritance. 1/3 of cases are new mutations [1]. This genetic disorder caused by mutations of the NF1 gene which is located on chromosome 17(17q11.2) [1]. Patients with NF1 may present with a variety of central nervous system complaints, such as seizures, learning disability and attention-deficit disorder. Intracranial lesions associated with NF1 include optic gliomas, sphenoid wing dysplasia, “unidentified bright objects” (UBOs) [1,2]. Cerebrovascular lesions such as moyamoya syndrome are rarely seen in NF1 [2]. Herein, we report of a 5-year-old female with NF1 and moyamoya syndrome and we briefly review the existing literature.

## Case report

**Figure 1:**
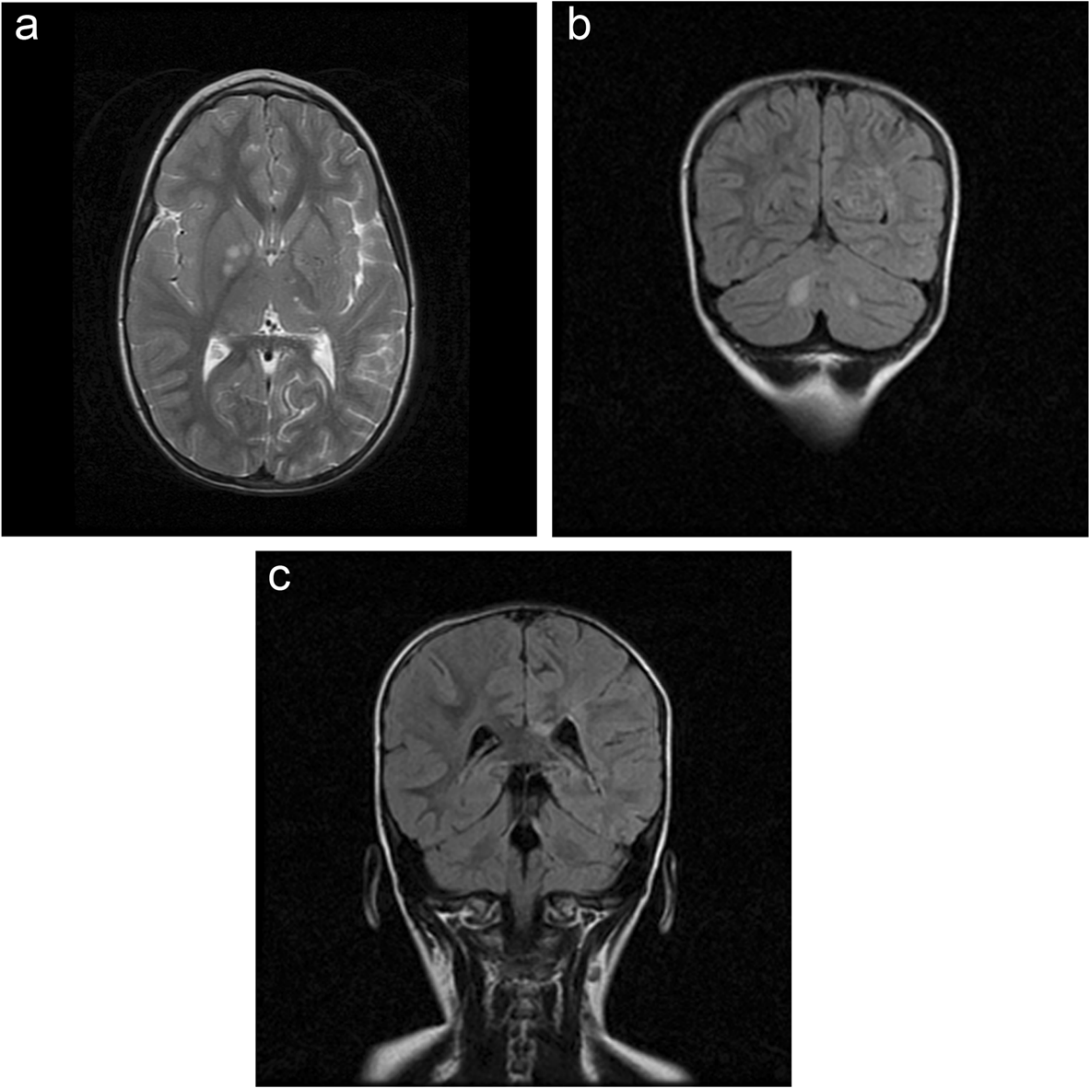
Brain MRI: T2-weighted images (TR/TE 4000/99) showed pathologic areas of increased signal, a. in right basal ganglia (UBO’S), b. Iin the deep white matter of cerebellum, c. in corpus callosum demonstrated typical areas of UBO’S (unidentified bright objects) in NF 1 disease.

**Figure 2:**
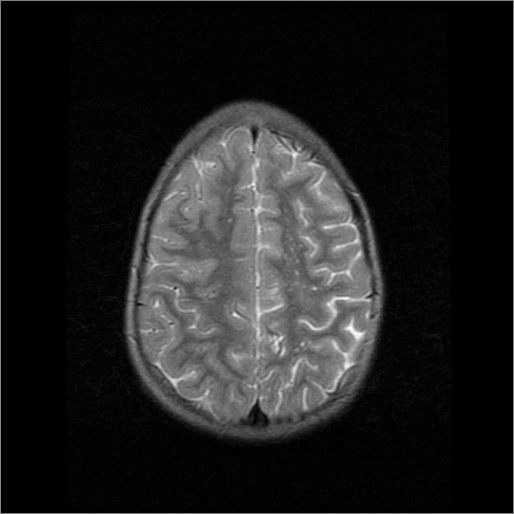
Brain MRI: T2-weighted images (TR/TE 4000/99) showed multiple sites empty signal mainly in the left basal ganglia and ipsilateral lunate center in as in the presence of collateral vessels.

**Figure 3:**
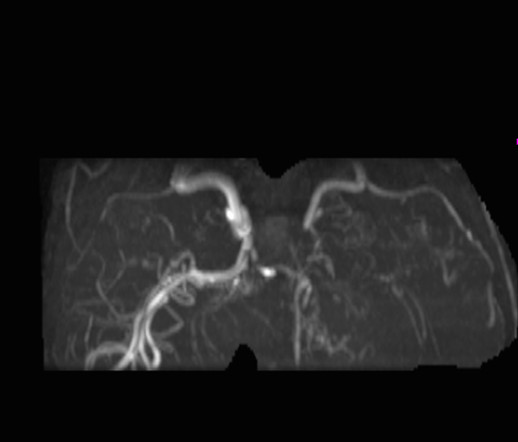
MR angiography of the brain (MRA): virgate imaging petrous segment and the proximal portion of the left internal carotid artery and occlusion of the distal vessel.

A 5 year-old female was admitted to the hospital with a history of acute right-sided weakness. The physical examination revealed several café-au-lait spots (larger than 0.5 cm) and freckling in the inguinal area and the axillae, thus fulfilling diagnostic criteria for NF1 [1,2]. The neurologic examination demonstrated right spastic hemiplegia with increased muscle tone of upper and low extremities, increased tendon reflexes as well as right facial nerve palsy.Regarding the NF1 work-up, including laboratory investigations, complete blood cell count, blood biochemistry, radiographs of long bones, electroencephalogram, visual and brainstem auditory evoked potentials, echocardiography, abdominal ultrasound and ophthalmologic examination were normal. Brain magnetic resonance imaging (MRI) of the brain, showed multiple small foci of T2 prolongation in the right periventricular white matter, as well as both globus pallidi and cerebellar hemispheres (“unidentified bright objects” -UBOs). These lesions were hypointense on T1-weighted images, did not show any mass effect, and did not enhance after intravenous contrast injection (Figure 1). Also, multiple dot-like flow-void enhancing areas in basal gaglia and thalamus representing lenticulostriade collateral vessels were seen (Figure 2).Magnetic resonance angiography of the brain (MRA) (and subsequent digital subtraction angiography), showed occlusion of the terminal left internal carotid artery (ICA) and severe stenosis of the proximal middle and anterior cerebral arteries, with multiple tiny basal collateral arteries (Figure 3). Anterior circulation was supplied mainly by these lenticulostriate and thalomo-perforator collaterals vessels. The right ICA and the posterior circulation were normal. These findings were considered as typical for moyamoya syndrome. Additional examinations included clotting, thrombophilia screen, iron and folate screen, demonstrated normal findings.

After neurosurgical consultation, surgical revascularisation [namely a left encephalo-duroarterio-synangiosis (EDAS)], was carry out electively. The intraoperative and postoperative course was unremarkable. Treatment with aspirin was immediately started and physical and occupational therapy was initiated. At 2-years follow-up her facial weakness had resolved (since 6 months postoperative), but still demonstrates a mild right hemiparesis.

## Discussion

NF1 is multisystemic disease, which affect the skin, central nervous system and bone system [1,2]. Its diagnosis is based on the clinical criteria established by the National Institutes of Health (NIH) Consensus Development Conference [3]. For a definitive diagnosis of NF-1, two or more of the following clinical characteristics must be present, six or more café-au-lait spots with a diameter more than 5 mm, two or more neurofibromas, axillary or inguinal freckling, one reticular neurofibroma, two or more Lisch nodules (hamartomas), an optic glioma, skeletal abnormalities, such as sphenoid dysplasia or pseudarthrosis of the tibia and first degree relationship with a patient suffering from NF1 [3]. These criteria are not sensitive to very young children, since many of them may appear gradually over the years, while children over 10 years old who do not meet the criteria are unlikely to suffer from NF1 [3].

NF1 vasculopathy is a significant but under-recognized complication of the disease, affecting arterial and venous blood vessels of all sizes. Although the arterial system is most commonly affected, venous circulation may also be involved [2]. A variety of vascular lesions have been noted in patients with NF1, including occlusion, aneurysm, pseudoaneurysm, ectasia, stenosis, fistula and rupture [4,5]. According to bibliographic data, the internal carotid artery is most commonly affected, followed by middle and posterior cerebral artery. Although rare in childhood, there was extracranial vascular involvement mainly in the renal artery [5]. The main characteristic of vascular lesions in patients with NF-1 is occlusion of the lumen and hyperplasia of the intima wall. Based on microscopic evaluation of the affected vessels, it has been proposed that the vasculopathy of NF-1 patients results from abnormal neurofibromin function that leads to excessive proliferation of vascular smooth muscle cells during normal maintenance of the vessel. Conversely, even though neurofibromin is known to be expressed in vascular smooth muscle cells, little is known about its function relating to controlling endothelial cell proliferation [2,5,6].

The precise mechanisms involved in NF1, the pathogenesis of vasculopathy are poorly understood but are likely related to the function of neurofibromin, the protein product of the NF1 gene [6]. Neurofibromin has been identified in the endothelial layer of bovine and rodent cerebral and renal arteries, as well as the aorta. It has been hypothesized that the loss of neurofibromin expression in endothelial cells may somehow cause vascular smooth muscle cells to proliferate [6]. It has also been suggested that neurofibromin helps maintain the integrity of the endothelial cell layer, and if this integrity is lost because of aberrant neurofibromin, vascular smooth muscle cells could proliferate [2,6].

Moyamoya syndrome is a rare disorder developing by stenosis and occlusion of small anastomotic vessels in distal branches of bilateral internal carotid arteries [7,8]. Nonetheless, its etiology continues to be ill-defined. Moyamoya syndrome is more frequently observed in the Japanese population, with an estimated incidence of one new case per 1,000,000 individuals per year [7]. The clinical findings present with neurological symptoms, whereas ischemic stroke develops in young adults, subarachnoid hemorrhage develops in older patients in moyamoya syndrome [7].

The prevalence of moyamoya syndrome in NF1 patients is estimated at 0.6%, with more than one hundred cases reported in paediatric patients since 1976 (Table 1) [8-43]. Moyamoya syndrome in patients with NF1 is often initially unilateral and often involves anterior vascular territories [43]. Most cases are asymptomatic, however, subsequent clinical and radiologic worsening is likely to occur [40-43]. In children, the clinical debut often implies ischemic events, such as transient ischemic attacks and ischemic infarcts, as well as focal seizures with headache, and intracranial hemorrhage might also be associated [40-43]. When symptoms are present, they include neurological findings such as paresthesia, headache, epileptic seizures, hemianopsia, nystagmus, aphasia, dysphasia and borderline mental level [16,40-43]. There are several lines of evidence indicating that moyamoya syndrome is related to genetic factors in familial cases. The gene abnormality has been detected in chromosome 17q25.2 [44]. NF1 gene is also mapped on chromosome 17q11.2 [2]. Hence, association of NF1 and moyamoya syndrome could be justified by close proximity neighboring of the responsible genes on chromosome 17 [10]. Moyamoya syndrome in NF1 patients is unilateral in up to 30% of cases, but the observation that progression to bilateral disease occurs in 10%–100% of these patients means that our patient may require long-term surveillance, and noninvasive perfusion studies may be helpful for assessment [9,45].

**Table 1:** Moyamoya syndrome in NF1 paediatric patients since 1976

**Authors**	**Year**	**Study**	**N (patients)**	**Age**	**Patients with moyamoya-NF1**
Duat-Rodríguez A et al. [42]	2014	Retrospective review	168		4
Partha S. G et al. [40]	2013	Retrospective review	312		7
Kaas B et al. [43]	2013	Retrospective review	187		5
Ghosh, P. S., et al. [10]	2012		398		15/312 (4,8%)
Luiz G D et al. [41]	2011	Case report	1	8 months	1
Ullrich, N. J. [11]	2011	Case report			1
Smith, M [12]	2011	Case report		18 m boy	1
Lin, N., et al. [13]	2011	Retrospective review 1988-2010	418	9.1y (1-21y)	13
Horiguchi, S., et al. [14]	2011	Case report	1	32y	1
Darrigo Junior, et al. [15]	2011	Case report		8 m female	1
King, J. A., et al. [16]	2010	Retrospective review (May 1996-December 2008)	33	< 16y	3
Tan, R. M., S. M. Chng, et al. [17]	2008	Case report	1	7y female	1
Koc, F., et al. [18]	2008	Case report	1	20y female	1
Borhani-Haghighi, A. and B. Sabayan [19]	2008				
Wintermark, P., et al. [20]	2007	Case report			1
Ullrich, N. J., et al. [21]	2007				
Pascual-Castroviejo, I., et al. [22]	2006		12		1
Desai, S. S., et al. [23]	2006	Review 1967-2002	54		14
Fujimura, T., et al. [24]	2004	Case report		15y female	
Scott, R. M., J. L. Smith, et al. [41]	2004	Retrospective review	143	<20y	13
Hug, E. B., et al.[25]	2002	Retrospective review Sep 1991-Aug 1997	27		1
El-Koussy, M., et al. [26]	2002	Case report			1
Rodriguez-Jadraque, R., et al. [27]	2000	Case report		9y	1
Serdaroglu, A., et al. [20]	2000	Case report		4y female	1
Piovesan, E. J., et al. [28]	1999	Case report		51y	1
Fujimoto, K., et al. [29]	1999	Case report		49y female	1
Siqueira Neto, J. I., et al. [30]	1998	Case report		28y male	1
Hattori, S., et al. [31]	1998	Case report		58y female	1
Barrall, J. et al. [32]	1996	Case report		19 m male	1
Gorrotxategi, P., et al. [33]	1994	Case report		4y male	1
Kestle, J. R., et al. [34]	1994	Retrospective review 1971-1990	47		3
Woody, R. C., et al. [35]	1992	Case report	1	3 m male	1
Sobata, E., H. Ohkuma, et al. [36]	1988	Case report	1	28y female	1
Gracia, C. M., et al. [37]	1986	Case report	1	33y female	1
Quest, D. O. et al. [38]	1985	Retrospective review	17		1
Sasaki, O., et al. [39]	1984	Case report	2	38y male	2
				29y female	

The diagnosis of moyamoya syndrome based on neuroimaging control. In patients with moyamoya syndrome, the brain computed tomography (CT) scan frequently shows ischemic and hemorrhagic lesions, although MRI is more helpful in the diagnosis of moyamoya syndrome because the provide greater parenchymal detail [46]. MRA is very useful for diagnosing moyamoya syndrome, with previous studies showing a sensitivity of 73% and a specificity of 100%. Sensitivity increases to 92% when MRA is combined with MRI findings [46]. The diagnosis of moyamoya syndrome in our patient was supported with MRI and MRA, which is standard practice in our clinical routine screening of children with NF1.

Most patients with NF-1 associated with vascular lesions are asymptomatic. Early diagnosis and appropriate surgical management are of utmost importance, to improve cerebral hemodynamics and reduce the incidence of subsequent ischemic events. However, close monitoring of these abnormalities is warranted because the long-term outcome of these vascular lesions is unknown. Moreover, early recognition of a cerebral vasculopathy may have helped prevent complications in several of these children [8,47,48].

Surgical intervention has become the treatment of choice for patients with MMS, and particularly surgical revascularization in order to increase the blood flow to the hypoperfused cortex [49-51]. On the index patient, the surgical procedure of choice was EDAS (encephaloduroarteriosynangiosis) and resulted in no further symptoms or brain insults.

In conclusion, vasculopathy in NF1 is a potentially serious and underestimated manifestation. MRA screening could be helpful in identified early vascular lesions in asymptomatic NF1 patients. Further studies are needed, in large cohorts of NF1 patients, to better understand the association between these conditions.

## Consent

Written informed consent was obtained from the patient’s guardian/parent/next of kin for the publication of this report and any accompanying images.

## Competing interests

The authors declare that they have no competing interests.

## Authors’ contribution

EV acquiered, analysed and interpreted the data and drafted the biggest part of the manuscript. ES: acquired the data and drafted a small part of the manuscript. DS: acquired the data. SB: acquiered the data and critically revised the manuscript. MK: acquired the data. AA: performed and interpreted the neuroradiological studies and critically revised the manuscript. DIZ: critically revised the manuscript for important intellectual content and supervised the study. All authors read and approved the final manuscript.
